# Exopolymer Diversity and the Role of Levan in *Bacillus subtilis* Biofilms

**DOI:** 10.1371/journal.pone.0062044

**Published:** 2013-04-26

**Authors:** Iztok Dogsa, Mojca Brloznik, David Stopar, Ines Mandic-Mulec

**Affiliations:** Department of Food Science and Technology, Biotechnical Faculty, University of Ljubljana, Ljubljana, Slovenia; Centre National de la Recherche Scientifique, Aix-Marseille Université, France

## Abstract

Exopolymeric substances (EPS) are important for biofilm formation and their chemical composition may influence biofilm properties. To explore these relationships the chemical composition of EPS from *Bacillus subtilis* NCIB 3610 biofilms grown in sucrose-rich (SYM) and sucrose-poor (MSgg and Czapek) media was studied. We observed marked differences in composition of EPS polymers isolated from all three biofilms or from spent media below the biofilms. The polysaccharide levan dominated the EPS of SYM grown biofilms, while EPS from biofilms grown in sucrose-poor media contained significant amounts of proteins and DNA in addition to polysaccharides. The EPS polymers differed also in size with very large polymers (Mw>2000 kDa) found only in biofilms, while small polymers (Mw<200 kD) dominated in the EPS isolated from spent media. Biofilms of the *eps* knockout were significantly thinner than those of the *tasA* knockout in all media. The biofilm defective phenotypes of *tasA* and *eps* mutants were, however, partially compensated in the sucrose-rich SYM medium. Sucrose supplementation of Czapek and MSgg media increased the thickness and stability of biofilms compared to non-supplemented controls. Since sucrose is essential for synthesis of levan and the presence of levan was confirmed in all biofilms grown in media containing sucrose, this study for the first time shows that levan, although not essential for biofilm formation, can be a structural and possibly stabilizing component of *B. subtilis* floating biofilms. In addition, we propose that this polysaccharide, when incorporated into the biofilm EPS, may also serve as a nutritional reserve.

## Introduction

It is generally accepted that the extracellular matrix, which holds constituent cells of the biofilm together, is composed of polysaccharides, proteins and nucleic acids [1]–[6]. These polymers, collectively known as exopolymeric substances (EPS), are only partially characterized in *B. subtilis* [2], [7]–[10], a bacterium, which is a model organism for the study of biofilm formation [11]–[14]. Furthermore, due to varying cultivation conditions, the use of different bacterial strains and EPS isolation procedures in different studies, the results from different groups are not directly comparable.

*Bacillus subtilis* floating biofilms (pellicles) that form at the air-liquid interphase have been most extensively studied in the MSgg medium using the undomesticated strain, *B. subtilis* NCIB 3610 [2], [11], [15]–[19]. The extracellular matrix of its biofilms is composed of exopolysaccharide produced by the *epsA-O* locus [15], [16], [20] and is comprised of glucose, galactose and N-acetyl-galactosamine [9]. In addition, *B. subtilis* secretes the 31-kDa TasA protein [21], which forms amyloid fibers that are proposed to mediate *Bacillus* cell-to-cell interactions and cell-to surface adhesion in conjunction with other extracellular components [22]. It is linked to the cell wall by TapA, a member of the *tapA-sipW-tasA* operon, which is a minor extracellular component and is presumably incorporated into the TasA fibers [16], [23]. Both TasA and polysaccharides encoded by *epsA-O* operon are considered to be essential for the formation of the *B. subtilis* NCIB 3610 floating biofilms grown in MSgg. The γ-polyglutamic acid (γ-PGA) is another component that can play an important role in *B. subtilis* biofilm formation and has been linked to mucoid appearance of the *B. subtilis* colonies [24], [25]. The production of γ-PGA is strain dependent and in pellicles formed by *B. subtilis* NCIB 3610 strain grown in γ-PGA stimulating medium the γ-PGA was not found [25]. Recently, the BslA (formerly YuaB) protein was shown to influence *B. subtilis* biofilm formation [18], playing an important role in biofilm surface repellency and microstructure [26], [27].

It is well known that *B. subtilis* synthesizes a non-charged extracellular polysaccharide, levan, when grown in batch cultures in sucrose-rich growth medium [28]–[32]. We therefore predict that levan may be present and play a role in biofilm matrix formation of *B. subtilis* biofilms in sucrose-rich growth medium. This has not been addressed previously. Levan is a β-(2→6) fructan biopolymer with occasional β-(2→1) branching and is considered to have a high potential for industrial application [33]. In sucrose-rich environments, *B. subtilis* (natto) Takahashi, a commercial natto starter, is able to selectively produce up to 50 g/L of extracellular levan during batch fermentation [28], [29]. The synthesis of levan is catalyzed by levansucrase (sucrose: 2,6-P-D-fructan 6-n- D-fructosyltransferase; E.C. 2.4.1.10) an extracellular enzyme with known crystal structure [34]. The structural gene of levansucrase, *sacB*, is part of *sacB–yveB–yveA* levasucrase tricistronic operon and is activated in the presence of sucrose [30], [32], [35], [36]. Sucrose activates an anti-terminator, SacY, which then allows the transcription of the *sacB* gene. In addition to SacY pleitropic regulatory genes *degS*/*degU*, *degQ* and *degR* also affect expression of *sacB* [36]. We searched 12 complete genome sequences of *B. subtilis* that are currently deposited in NCBI database for the levansucrase gene (*sacB*), and all carried a gene with a high homology(>95%) to *B. subtilis* 168 *sacB*, which was extensively used in other levan studies. This *sacB* gene was also detected in *B. subtilis* NCBI 3610 (100% homology) used in this study, suggesting a potential of this strain for levan synthesis. Also, *B. subtilis* forms biofilms on plant roots [37]–[39], where sucrose can be found [40], and may there induce the synthesis of levan. It is therefore important to address whether levan is present in the *B. subtilis* biofilm matrix in addition to extracellular *epsA-O* polysaccharides and TasA protein. We speculate that levan is an integral part of the biofilm matrix, where it may interact with other biofilm polymers and affect the biofilm phenotype.

In this study we explored EPS diversity of *Bacillus subtilis* NCIB 3610 biofilms in sucrose-rich and -poor media, including the possible presence and the role of levan in the biofilm matrix. We have taken the advantage of the fact that the expression of levansucrase (*sacB*), an enzyme that catalyzes the polymerization of levan, is induced by the presence of sucrose in the medium [30], [32], [35], [36]. In this respect biofilms grown in a sucrose-rich medium (SYM) were compared with the biofilms formed in two sucrose-poor growth media; glycerol and glutamate-based growth medium (MSgg), or glucose-based minimal growth medium (Czapek). In addition, experiments were conducted where sucrose was added to MSgg and Czapek growth medium. The three media were chosen, because they have been exploited previously to study *B. subtilis* EPS [7], [11], [41], but not in systematic way or for exploration of the biofilm EPS. We characterized the contribution of sucrose to the *B. subtilis* biofilm and show for the first time that in the presence of sucrose levan is a part of the biofilm matrix, where it contributes to biofilm robustness. Furthermore, we present the finding that composition of EPS in the biofilm and in the condition medium below the biofilm is different and changes with growth medium.

## Materials and Methods

### Bacterial strains, growth media and statistical analysis

Strains used in this study are the wild *Bacillus subtilis* NCIB 3610 (undomesticated prototroph) [42] and its derivatives: the *eps* mutant ((*epsA-O*)*::tet*), the *tasA* mutant (*tasA::spc*) and the *tasA eps* double mutant (*tasA::spc* (*epsA-O*)*::tet*). The strains were kindly provided by R. Kolter [2]. In addition, *B. subtilis* YC164 (P*_epsA_*-*gfp* at the *amy* locus in 3610, Cm^R^) strain was kindly provided by Y. Chai and R. Losick [20]. The overnight cultures were prepared in 25 mL LB medium and incubated at 28°C with shaking at 200 rpm for 13.5 h. Cultures were then inoculated into liquid SYM, Czapek or MSgg medium with the initial OD_650_ = 0.03 a.u.. 55 mL and 270 µL/well of growth media were used for experiments in petri dishes and microtiter plates, respectively. The sucrose based SYM, glucose rich, Czapek and glycerol and glutamate MSgg media compositions were prepared as given by Shida et al. [41], Morita et al. [7] and Branda et al. [11], respectively. SYM is composed of (w/v) K_2_HPO_4_ (1.2%), KH_2_PO_4_ (0.4%), (NH_4_)_2_SO_4_ (0.3%), MgSO_4_ (0.6%), MnSO_4_ (0.00015%), ammonium iron (III) citrate (0.002%), yeast extract (2%), sucrose (20%); Czapek of glucose (3%), NaNO_3_ (0.3%), yeast extract (0.01%), K_2_HPO_4_ (0.05%), MgSO_4_·7H_2_O (0.05%), FeSO_4_·7H_2_O (0.001%) in MnSO_4_·nH_2_O (0.0005%); MSgg of MOPS (morpholinepropane sulfonic acid) (2.1%), glycerol as a carbon source (0.5%), glutamate (0.5%), K_3_PO_4_ (0.1%), tryptophan (0.005%), phenylalanine (0.005%), MgCl_2_ (0.02%), CaCl_2_ (0.008%), FeCl_3_ (0.0008%), MnCl_2_ (0.0006%), thiamine (0.00006%), ZnCl_2_ (0.000014%). The influence of sucrose on biofilm formation was studied by adding sucrose to the Czapek and MSgg medium in the petri dishes to the same concentration as in SYM medium (i.e. 200 g/L).

Statistical analysis was performed in all cases where the treatment differences were small compared to experimental error. The student's t-test was applied without assuming equal variances.

### Biofilm development, morphology and thickness

Wild type biofilms were cultivated as standing cultures in microtiter plates for 8 days at 37°C and macromorphology and micromorphology were monitored daily. Macromorphological properties were determined using a stereomicroscope (Leica Wild M10) equipped with a digital camera Nikon D40. Micromorphology and eventual presence of spores were studied using an inverted microscope Zeiss Axio Observer Z1, equipped with phase and differential interference contrast (DIC) [43]. In addition, optical density at 650 nm (OD_650_) of *B. subtilis* standing cultures in microtiter plates [25] was measured by Thermo Multiscan Spektrum plate-reader.

The morphology and thickness of *B. subtilis* biofilms were monitored in petri dishes (diameter, 8.7 cm) by growing wt, *tasA*, *eps* and *tasA eps* strains for 24 h without shaking at 37°C. The ratio of biofilm surface to volume of the medium in the petri dishes was the same as in microtiter plates. To determine biofilm thickness a piece of biofilm was placed inside a silicone rectangular frame (height: ∼2 mm) on a microscope slide that prevented the biofilm from flattening after it was covered by a coverslip. The thickness was determined microscopically by measuring the difference in height between the bottom and the top of the biofilm [44], [45] using digital z-axis positioning. For this purpose the inverted microscope Zeiss Axio Observer Z1 operated by software Axiovision Rel. 4.8 was used.

### EPS isolation

Biofilms were grown in 55 mL of liquid growth medium (SYM, Czapek, MSgg), inoculated with wt, *tasA*, *eps* and *tasA eps B. subtilis* strains in petri dishes at 37°C. The sucrose-supplemented Czapek and MSgg were inoculated with wt only. Controls consisted of petri dishes with non-inoculated growth media. After incubation for 24 h, the biofilm and spent medium were separated by carefully transferring the latter to a fresh centrifuge tube. The remaining biofilm was also transferred to a fresh centrifuge tube and both samples were diluted by PBS buffer to 1∶1 ratio, vortex stirred, and distributed to Eppendorf tubes. Samples were then sonicated with an MSE 150 Watt Ultrasonic Disintegrator Mk2 with exponential probe for 5 s at amplitude 15 µm. Microscopic examination verified that this process did not cause significant lysis of *B. subtilis* cells, which is consistent with the observations of Branda et al. [2]. After sonication the samples were pooled and 0.2 M NaOH was added to a final concentration of 0.1 M and incubated for 10 min at room temperature with short intermittent vortex stirring after 5 min and at the end of incubation. This step increases the charge on the EPS, thus increasing its solubility, and is often applied to isolate EPS from sludge biofilms, where it is considered not to cause significant cell lysis [46]–[50]. Consistently, microscopic examination did not show any substantial cell lysis. Samples were then chilled on ice for 5 min before adding cold 0.4 M HCl to a final concentration of 0.1 M, which neutralized the sample. Cell mass was separated from supernatant EPS by centrifugation (10 000 g, 10 min, 4°C) and supernatant was transferred to a 3-fold volume of 96% cold EtOH [29] and left at 4°C for 20 h to precipitate EPS. The cell pellet was dried overnight at 55°C and the precipitated EPS was collected by centrifugation (10 000 g, 10 min, 4°C) and re-dissolved in deionized water in a 1∶10 ratio. NaCl was then added to a final concentration of 0.5% and EPS was re-precipitated by adding 3 volumes of 96% cold EtOH, followed by incubation at 4°C for 20 h [8]. EPS was again collected by centrifugation (10 000 g, 10 min, 4°C) and dried at 55°C. Stock aqueous solutions of EPS were prepared and stored at −20°C prior to further analysis.

### Chemical analysis of isolated EPS

The polysaccharide content of EPS was determined using the general phenol sulphuric acid method [28], [51]–[55] with the modifications of Cuesta et al. [56], except that samples were incubated for 20 min at 100°C. Total protein content in EPS samples was determined by the Bradford method [48], [57], [58] after dilution of EPS in 0.1 M NaOH [59]. The EPS nucleic acid content was determined spectrophotometrically (Thermo Scientific Nanodrop 1000). To ensure better EPS solubility, samples were prepared in 0.05 M NaOH. However, at alkaline pH the UV absorption properties of proteins and nucleic acids are different than in distilled water, which is usually taken as a solute for nucleic acid or protein samples. Therefore, we prepared calibration curves of the absorbance at 260 and 280 nm, with salmon sperm DNA taken as a nucleic acid standard and BSA (bovine serum albumin) as a protein standard. In this way molar extinction coefficients (k_Prot,280_; k_NA,280_; k_Prot,260_; k_NA,260_) were determined and concentrations calculated from equations using standard equations for determination of nucleic acid concentration [60]:
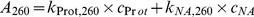
(1)

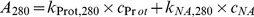
(2)


The fractions of individual EPS constituents i.e. polysaccharides, proteins and nucleic acids where determined by dividing the mass of individual constituent by the sum of masses of all constituents.

### High performance size exclusion chromatography (HPSEC) analysis of EPS

The molecular weight distribution of isolated EPS was determined using high performance size exclusion chromatography (HPSEC), which utilized an isocratic pump (Knauer K-501, Germany), a Refractive Index (RI) detector (Knauer 2300), a UV detector (Knauer K-2501) and a thermostated column compartment (Colum-thermostated Knauer Jet stream 2 plus). Separation was performed using successively linked PSS Suprema Analytical 10 000 Å, 1000 Å, 100 Å columns (300 mm×8 mm, 10 µm particle size), with polyhydroxy metacrylate polymer as a stationary phase. These columns tolerate pHs from 1.5 to 13 and temperatures up to 80°C. The column and detector flow cell temperatures were maintained at 35°C. Separation was achieved using 0.05 M NaOH as a mobile phase at a flow rate of 1 mL/min. The molar mass distributions of EPS were determined using a calibration curve prepared with dextran standards of peak molecular weight (Mp) from 180 Da to 401 kDa (Polymer Standard Service, Mainz, Germany). The injection volume was 20 µm and prior to injection NaOH was added to EPS samples to obtain the final concentration of the mobile phase (0.05 M NaOH). The chemical composition of HPLC fractions was inferred by taking into account the UV_260_, UV_280_ and RI chromatograms. Only data with a RI signal to noise ratio of at least 5 were considered. Three calibration curves of dextran, BSA and salmon sperm DNA were used to determine the extinction coefficients for polysaccharides, proteins and nucleic acids, respectively. In addition to eq 1 and eq 2, a third equation, which relates the concentrations of polysaccharides, proteins and nucleic acids to the Refractive index (RI) detector signal, was used: 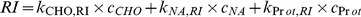
(3)


Estimates of concentrations of polysaccharides, proteins and nucleic acids (c_CHO_, c_Prot._, c_NK_) were obtained by solving the linear system of three equations (Eq 1, 2 and 3). Due to the typical absence of strong chromophores, the polysaccharides absorb UV in the experimental range very weakly, especially in comparison to nucleic acids and proteins. For example, UV_280_ absorption of standards in our experiments differed in orders of magnitude (k_NA_∶ k_Prot._ ∶ k_CHO_≈1000∶100∶1). In contrast, RI changes induced by polysaccharides, proteins or nucleic acids were similar.

### Shear stress stability of biofilms

After 9 days of incubation 50 µL of biofilm suspensions was mixed with PBS in a ratio 1 to 10 in a 1.5 mL Eppendorf tube. Tubes were shaken vigorously by a vortex stirrer at 1500 rpm for 1 min and 10 µL of vortex stirred suspension was used for slide preparations that were examined in the dark field mode of the stereomicroscope (Leica Wild M10).

### TLC analysis of EPS

The sugar composition of exopolysaccharides was determined using thin layer chromatography (TLC). EPS (0.5–1.5 mg/mL) from *B. subtilis* was hydrolysed with 3% (v/v) trichloroacetic acid at 55°C for 15 min and then neutralized with 1 M NaOH. This procedure takes advantage of the fact that levan is composed solely of fructose, enabling determination of the identity of levan [61], [62]. Levan (0.5 g/L) from *Erwinia herbicola* (Sigma-Aldrich) was used for comparison. Samples were loaded on a TLC silica gel 60 RP-18 F_254S_ (Merck) plate and chromatography was performed in the solvent system consisting of acetone and milliQ water in a 9∶1 ratio. Sugars were visualized by spraying a detection solution (composed of 0.2 mL (100%) aniline, 0.2 g diphenylamine, 20 mL acetone and 2 mL 85% phosphoric acid), and incubating at 120°C for 20 min.

### Expression of epsA-O operon

Expression of the *epsA-O* operon was evaluated by quantitative fluorescence microscopy using strain *B. subtilis* YC164. The *B. subtilis* NCIB 3610 wild type strain was used to determine autofluorescence. The biofilms were collected, and washed twice by centrifugation at 10000 rcf for 5 min with 0.5 mL of PBS buffer. Cells were immobilized on 10-well poly-L-lysine covered diagnostic slides (Thermo-Scientific) and the anti-fading agent Slow-fade (Invitrogen) was applied. Fluorescence images were taken by Zeiss Axio Observer Z1 with 100x/1.40 Oil objective “Plan-apochromat” and equipped with AxioCam MRm Rev.3 camera; the fluorescent light source was HBO 100 Illuminator. For excitation and emission 38HE filter set was used. The flat-field correction and normalization was performed using sodium fluorescein (0.75 g/mL) as a standard [63]. The captured images were analyzed with ImageJ (1.43u) software. Prior to fluorescence calculation artifact objects were removed manually. Weighted average of mean normalized intensities of fluorescent objects was calculated for individual images. For each time point 500–1000 cells were evaluated for their fluorescence.

## Results

### Architecture of *B. subtilis* biofilms in sucrose-rich and poor growth media

**Figure 1:**
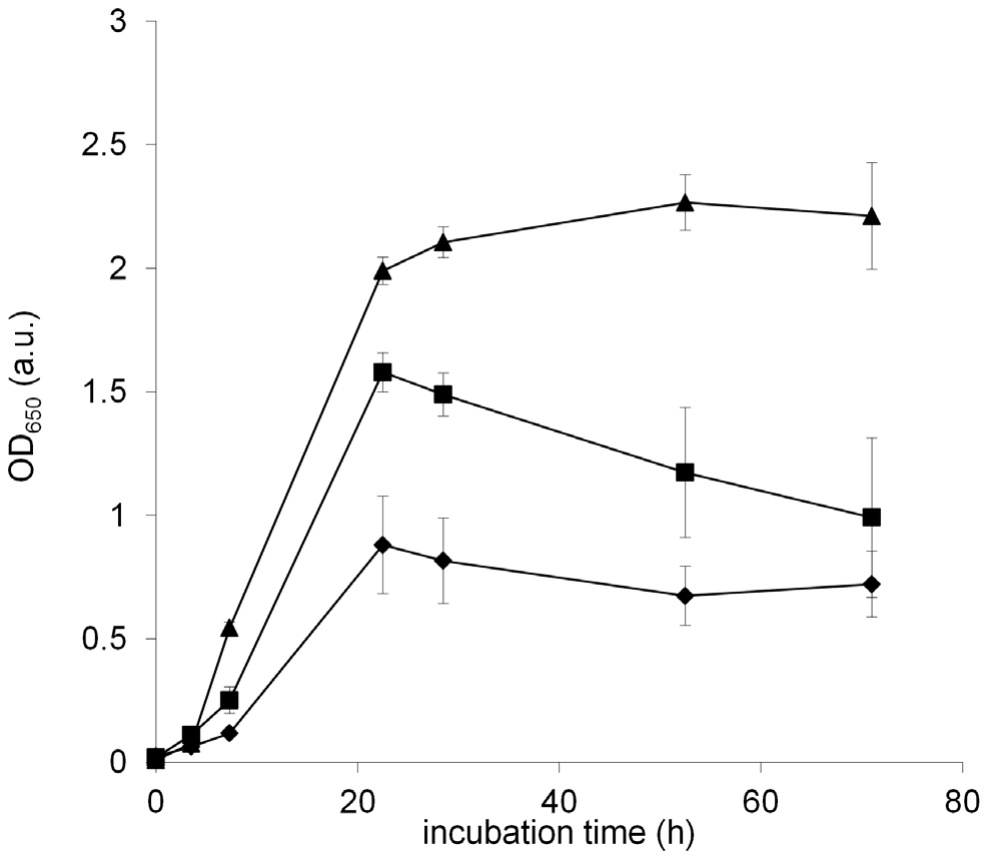
Optical densities (OD_650_) of standing cultures of *B. subtilis* grown in SYM (▴), Czapek (▪) and MSgg (♦). One of the four replicates is shown, with error bars representing standard deviations.

The growth of *B. subtilis* standing cultures was monitored in three media (SYM, Czapek and MSgg) during incubation for 70 h (Fig. 1). Growth rates and final OD_650_ values significantly differed in all three media with the highest growth rate observed in SYM medium (t_gen_ = 1.6 h±0.4 h; p<0.005), followed by Czapek (t_gen_ 3.0 h±0.2 h; p<0.0005) and MSgg (t_gen_ 4.1 h±0.2 h). However, the maximal OD_650_ in the three media was reached at approximately the same time (around 24 h) after which the growth leveled off or decreased (Czapek). This suggested that the floating biofilms entered stationary phase at similar time in all three media. These biofilms were considered to be mature after this time.

**Figure 2:**
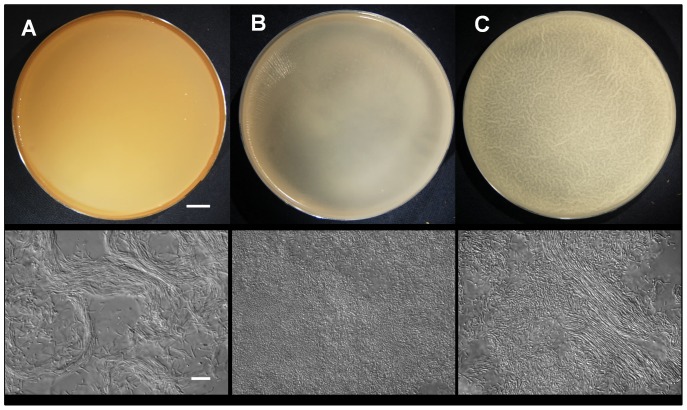
Macroscopic (top row) and microscopic (bottom row) images of biofilms (pellicles) grown for 24 h at 37°C in petri dishes in three different media. (A) SYM, (B) Czapek and (C) MSgg. Scale bar represents 10 mm (top row) and 10 µm (bottom row).

The mature biofilms differed in pigmentation and architecture (Fig. 2), as reflected in a different surface texture and thickness of the biofilm (Table 1). Biofilms in SYM and Czapek growth medium were smooth, whereas the surface of MSgg biofilms appeared wrinkled. Microscopic observation indicated two different morphologies; cells were organized in long parallel chains in MSgg and in SYM biofilms, or in densely packed short rods with random orientation in Czapek biofilms. The biofilm thickness of the wild type strain was the largest in SYM (500 µm±100 µm) (Table 1). In SYM growth medium floating biofilms formed after incubation for 5 h and spores were microscopically detected after 120 h. In Czapek growth medium biofilms formed after 23 h with spores appearing already after 46 h. Similarly, in MSgg growth medium biofilms formed after 23 h, but spores were detected even earlier, after 29 h. In addition, yellow pigmentation developed after 75 h.

**Table 1:** Biofilm (pellicle) thickness in µm.

strain	SYM	Czapek	MSgg
wt	500±100	20±5^*^	230±30^†^
*eps*	15±5^*^	Nd^a^	Nd^a^
*tasA*	170±90^†^	18±1^*^	35±5
*tasA eps*	Nd^a^	Nd^a^	Nd^a^

The error bars represent standard deviation of the mean (n≥4).

a Not detectable

*, ^†^Among these pairs of values the difference is insignificant (p>0.05)

The cell biomass and mass of EPS were determined in the floating biofilms and in spent media underneath biofilms (Table 2). In MSgg growth medium most of the cell biomass was located in the biofilm. The ratio of cells in the biofilm to cells in the spent medium was 73 to 1. The situation was very different in Czapek growth medium, where most of the cells remained in the spent medium (the ratio was 0.3). In SYM the ratio was close to 1, indicating the cells were equally distributed among both phases. EPS was detected in the biofilm and in the spent medium in all three media. In MSgg growth medium EPS was approximately equally distributed between the biofilm and spent medium, while in Czapek more EPS remained in the spent medium. This was even more pronounced in SYM, where approximately 80% of the EPS remained in the spent medium. Overall, total EPS production was significantly higher in SYM medium than in MSgg and Czapek (i.e. 290- and 760-fold more, respectively). Biofilm EPS concentration was also highest in SYM, (130±30) mg of EPS/mL biofilm; in Czapek and MSgg EPS concentrations were (6±4) and (2±1) mg of EPS/mL biofilm, respectively.

**Table 2:** Cell and EPS dry mass in biofilm and spent medium below the biofilm.

		SYM	Czapek	MSgg
Cell mass (mg):	biofilm	169±5	20±7	66±7^#^
	spent medium	130±30	60±30^#^	0.9±0.05^*^
EPS mass (mg):	biofilm	400±40	0.7±0.4^*^	3±2^*,†,‡^
	spent medium	1800±300	2.2±0.6^‡^	4.6±0.9^†^

Biofilms (pellicles) were grown in 55 mL of either SYM, Czapek or MSgg medium in petri dishes. Mass was measured in mg per petri dish. The error bars represent standard deviation of the mean (n≥4).

*, †,‡, # Among these pairs of values the difference is insignificant (p>0.05)

### Medium-dependent changes of *B. subtilis* EPS

**Figure 3:**
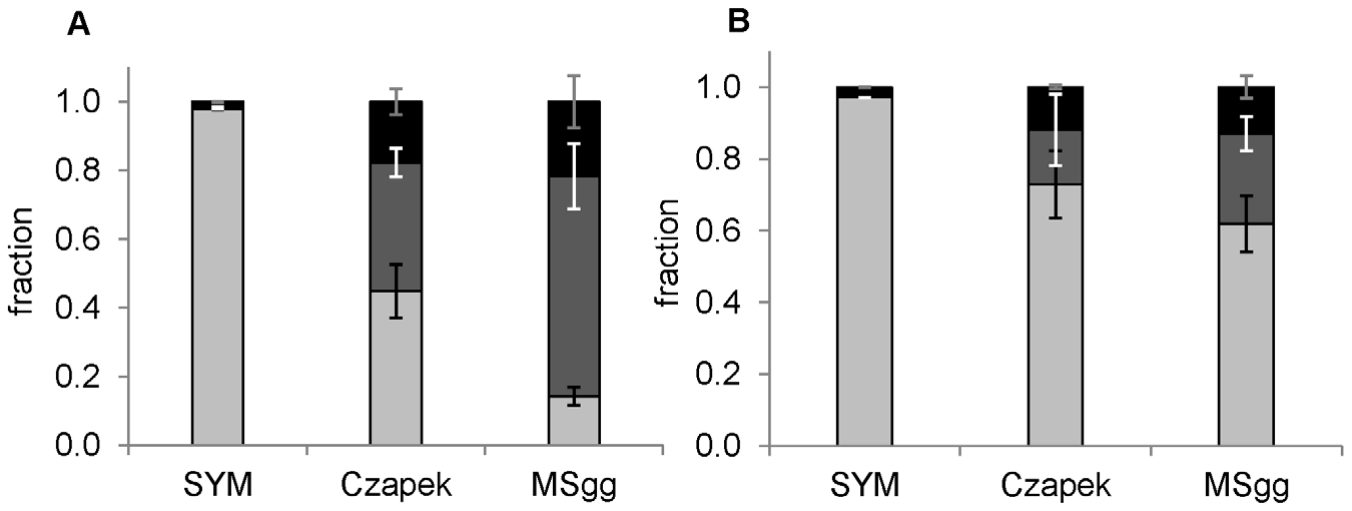
Fractions of polysaccharides (▪), proteins (▪) and nucleic acids (▪) in EPS isolated from biofilms (A) and corresponding spent medium below the biofilm (B) grown in three different media. SYM, Czapek, MSgg. The error bars represent standard deviation of the mean (n≥4).

The chemical composition of isolated EPS is given in Fig. 3. Although extracellular polysaccharides, proteins, and nucleic acids were produced in all growth media, concentrations of individual components differed significantly after incubation for 24 h. The EPS isolated from biofilms grown in sucrose-rich SYM growth medium mainly contained polysaccharides and only low amounts (<3%) of proteins and nucleic acids and a qualitatively similar composition of EPS was observed in the biofilm and in the spent medium. Polysaccharide content was much lower in biofilms grown in Czapek (p<0.01) and MSgg (p<0.001) and the distribution of the extracellular components differed between the biofilm and the spent medium. The chemical nature of polysaccharides isolated from biofilms was determined by TLC after acid hydrolysis of isolated EPS. Only polysaccharides isolated from SYM grown biofilms gave a single TLC band, corresponding to a monosaccharide fructose (Fig. S1). In contrast, no monosaccharides were detected in hydrolysates of EPS isolated from MSgg and Czapek media. These results confirmed that levan (fructan) is an integral component of SYM, but not of MSgg- and Czapek-grown biofilms, which presumably contain relatively acid resistant polysaccharides. It is well known that *B. subtilis* produces levan (fructan) in sucrose-rich medium [28]–[32], and our TLC results now confirmed its presence also in *B. subtilis* biofilms.

**Figure 4:**
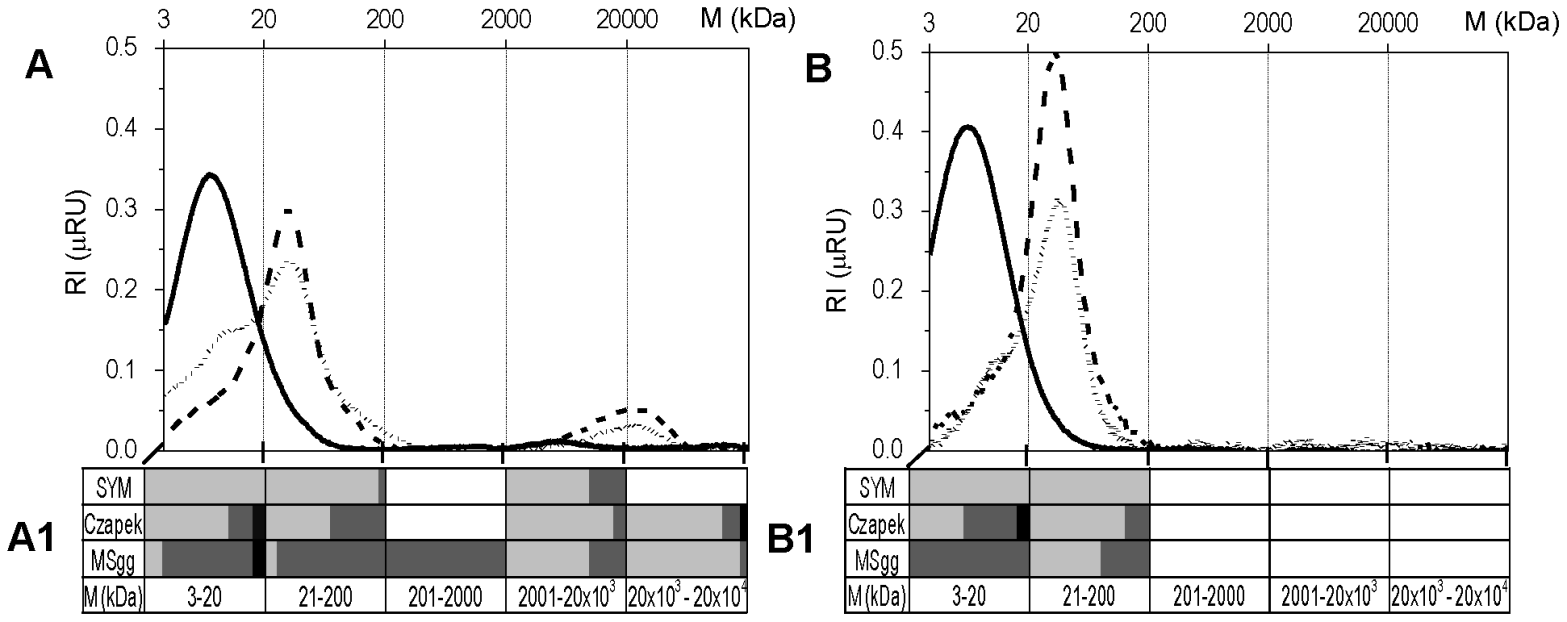
Size exclusion liquid chromatography (HPSEC) representative chromatograms of EPS isolated from biofilm (A) and from spent medium below the biofilms (B) grown in three media. SYM (—––); Czapek (– – –), and MSgg (- - - - - -). The molecular weight (M) distributions were graphically split into five size fractions. Their chemical composition and relative abundance of polysaccharides (▪), proteins (▪) and nucleic acids (▪) in these size fractions is graphically represented in A1 and B1. Chemical composition of EPS size fractions was determined from RI (refractive index) chromatograms shown in A and B and from UV 280 and 260 nm chromatograms (not shown).

The size distribution of EPS components isolated from biofilms and spent media is given in Fig. 4. The results from high performance size exclusion chromatography (HPSEC) indicated that isolated EPS components range in size from 3 to 100,000 kDa. Large components were present only in biofilms. In biofilms grown on sucrose-rich SYM medium, two well defined Gaussian peaks with Mp = 7 kDa and Mp = 5000 kDa were detected. The low molecular peak was composed of polysaccharides that were levans, based on TLC results. The high molecular peak was much smaller, indicating lower concentrations of its constituents; polysaccharides and proteins. EPS from biofilms grown in sucrose-poor Czapek and MSgg media produced a different pattern of HPSEC peaks. The low molecular peak indicated by a shoulder in Fig. 4A was slightly shifted toward a higher molecular weight compared to SYM chromatogram and was composed of nucleic acids, proteins and polysaccharides. A sharp peak at molecular weight (Mp) = 30 kDa contained polysaccharides and proteins but no nucleic acids. The last peak represented polymers of high molecular weight (Mp = 25000 kDa) and was also composed of only polysaccharides and proteins. Based on TLC results, however, none of the peaks contained levan (Fig. S1). The EPS composition in MSgg and Czapek spent media below the biofilms (Fig. 4B) was different than in the biofilm, most notably lacking the high weight molecular peak and the shoulder of the low molecular peak.

### Sucrose influences biofilm phenotypes of *B. subtilis*

In MSgg medium, similar biofilm defective phenotypes were observed for *eps* and *tasA* mutants as reported previously [2]. Both, the *eps* mutant and *tasA eps* double mutant failed to form biofilms, while the *tasA* mutant formed a weak biofilm of decreased thickness (Table 1, Fig. S2). In contrast, in sucrose-rich SYM medium the *eps* mutant still formed biofilm, albeit with a markedly reduced thickness compared to the wild type. Also the *tasA* biofilm defective phenotype was less pronounced in the presence of sucrose and only the *tasA eps* double mutation completely failed to form the biofilm. However, the double mutant was still secreting levan, which accumulated in the spent medium (data not shown) suggesting that levan alone is not sufficient for biofilm formation and that either the EpsA-O polymer or TasA need to be present for cells to form a pellicle.

**Figure 5:**
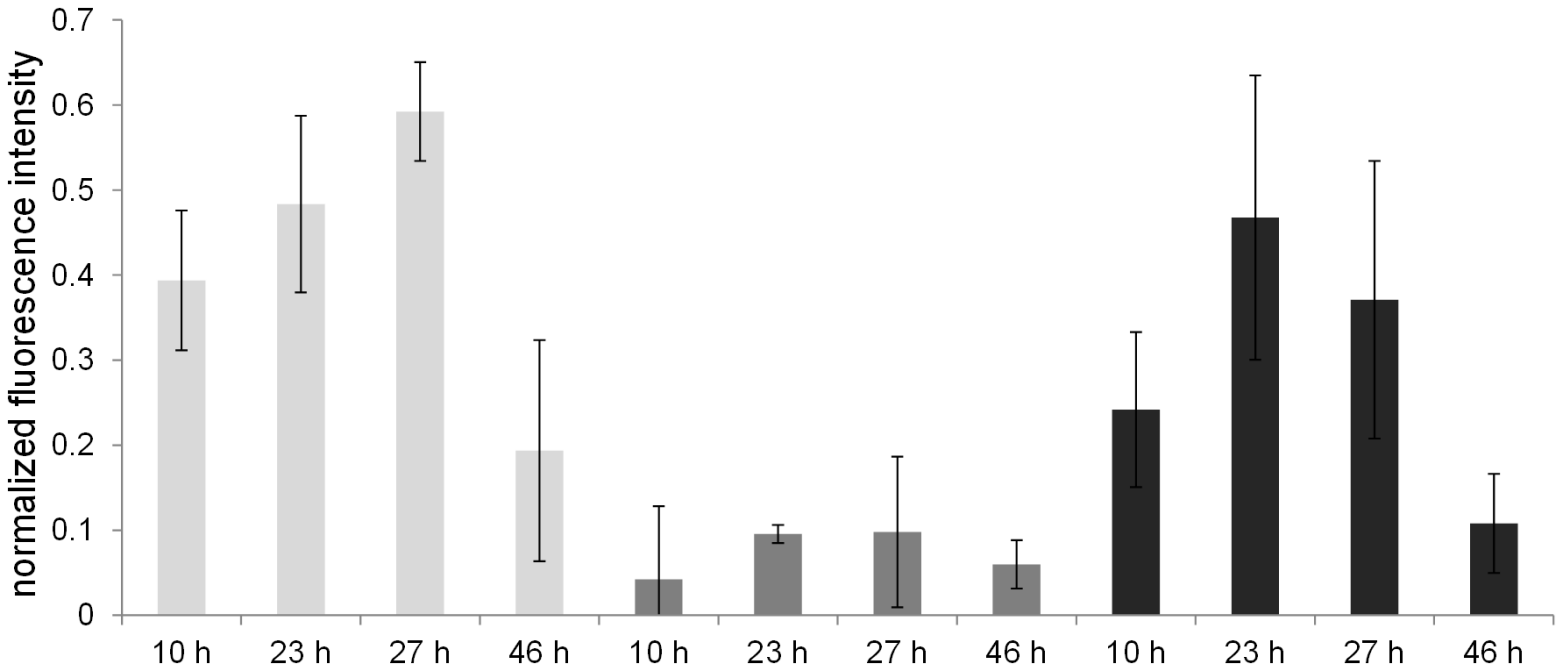
Expression of the *epsA-O-gfp* operon in biofilms grown in SYM (▪), Czapek (▪) and MSgg (▪) media given as weighted average of mean normalized fluorescence intensities of individual objects determined by fluorescence microscopy (n≥5).

**Figure 6:**
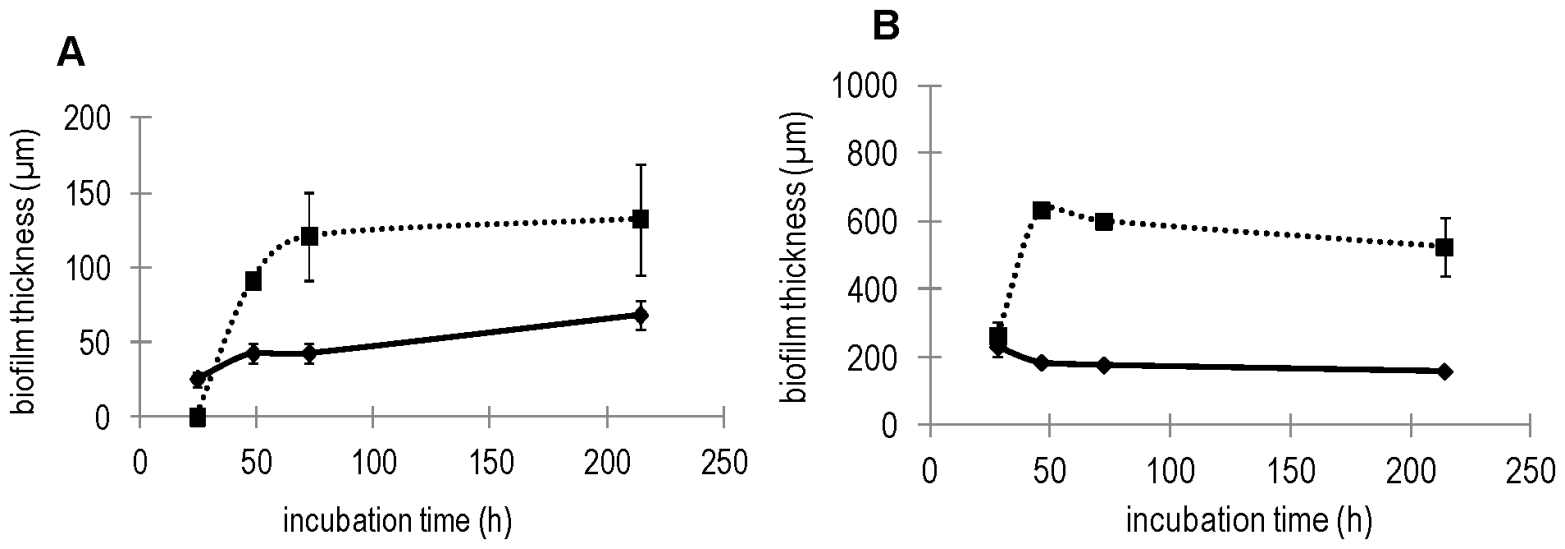
Biofilm (pellicle) thickness at different time points during growth in (A) Czapek (———), Czapek + sucrose (▪ ▪ ▪ ▪ ▪); (B) MSgg (———), MSgg + sucrose (▪ ▪ ▪ ▪ ▪). The errors represent standard deviation of the mean (n≥4).

The expression of *epsA-O* increased over time and was comparable in biofilms grown in SYM or MSgg, while on average much lower expression of this promoter was observed in biofilms grown in Czapek medium (p<0.05), (Fig. 5). The maximum expression of *epsA-O* in SYM and MSgg was estimated to arise at 20–30 h (p<0.05) and then decreased at 46 h. Addition of sucrose to MSgg and Czapek medium had a significant effect on biofilm formation and structure. As illustrated in Fig. 6, both the dynamics and the thickness of the biofilm changed compared to the controls with no sucrose. In Czapek medium supplemented with sucrose a delay of 24 h was observed before biofilm started to form. After that biofilm thickness increased rapidly and reached 140 µm, which is significantly higher than in the absence of sucrose. TLC analysis demonstrated that levan was indeed produced only in sucrose-rich Czapek and MSgg medium (Fig. S1). In MSgg medium the addition of sucrose also increased biofilm thickness up to 3-fold and the color and texture of biofilms in both MSgg and Czapek media supplemented with sucrose changed substantially (Fig. S3), becoming more similar to SYM (Fig. S4).

**Figure 7:**
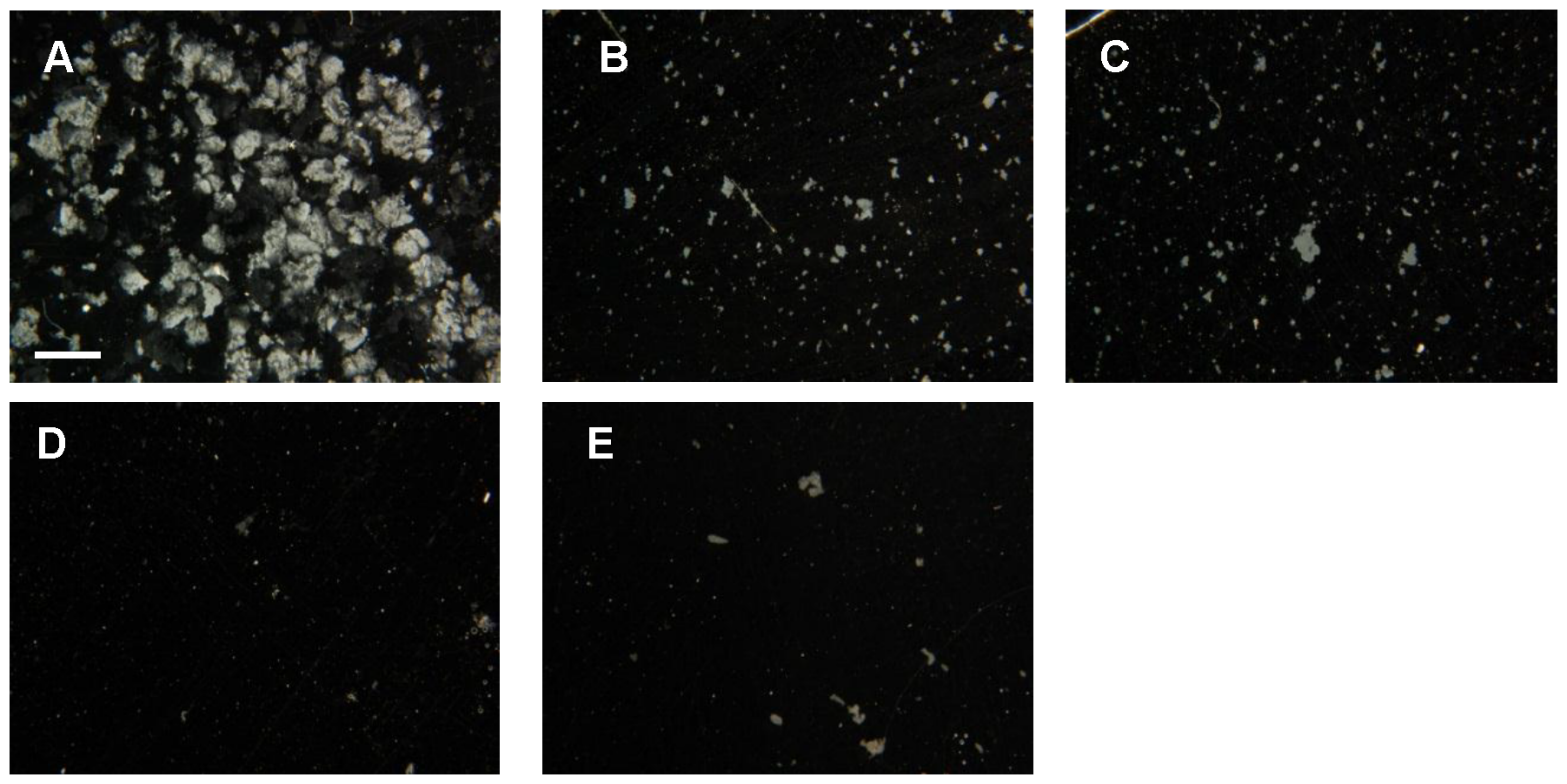
Images of 9-day old biofilms disintegrated by vortex stirring as observed under stereomicroscope. Scale bar corresponds to 1 mm. Biofilms from (A) MSgg + sucrose, (B) Czapek + sucrose, (C) SYM, (D) MSgg and (E) Czapek growth medium.

Next the stability of biofilms grown in different media and exposed to shear stress induced by stirring was tested qualitatively (Fig. 7). The biofilms of MSgg and Czapek medium appeared fragile, already at the time when they were transferred from the surface of the growth medium to the Eppendorf tube. Consistently, after vortex stirring, most of the biofilm constituents of MSgg and Czapek biofilms disintegrated into well dispersed cells, making them invisible for stereomicroscopy (resolution >4 µm). In contrast, biofilms grown in sucrose-rich media disintegrated into larger visible particles. This effect was especially pronounced in sucrose-supplemented MSgg medium, indicating that sucrose increased the mechanical stability of *Bacillus* biofilms.

## Discussion

In the present study *B. subtilis* the composition of EPS was determined in mature biofilms grown in three different media. A mature biofilm was defined as a biofilm that has developed most of its phenotypic properties; had reached maximal density, according to the OD_650_ measurements of the standing culture, and whose thickness remained approximately constant before the onset of sporulation. All biofilms achieved maturity at 24 h, which was associated with maximal expression of *epsA-O*, and at this time no sporulation was observed in any of the biofilms. The differences in absolute amounts of isolated EPS, in the EPS to cell ratio and in molecular weight distribution of EPS polymers in mature biofilms grown in different media for 24 h ranged over two orders of magnitude. Similarly, changes in EPS composition, isolated from the spent medium below the biofilms, were also observed. For example, a significant amount of a 7 kDa protein was detected only in biofilms implying that another extracellular protein component, besides TasA, which is 31 kDa in size, may be important for biofilm formation. In addition, the largest exopolysaccharides detected in *B. subtilis* biofilms thus far [14], [64] were found exclusively in the EPS of biofilms, but not in the spent media.

Biofilms and associated EPS have not been explored previously in sucrose-rich SYM medium, which is known to support high production of fructose-based polysaccharide levan in *B. subtilis*. We now found that this medium sustained the growth of robust biofilms, which were significantly thicker than those formed in MSgg or Czapek medium. Cells in SYM medium produced large amounts of levan, which was mostly located in the spent medium, but a significant fraction partitioned to the floating biofilm, representing more than 97% of its EPS components. Levan was also identified in biofilms grown in MSgg and Czapek supplemented by sucrose. Therefore these results for the first time point to levan as an important component of *B. subtilis* biofilm EPS. This is consistent with observations that levan is an important constituent of *Pseudomonas syringae* [53] and *Streptococcus mutans* [65] biofilms.

A biological role of levan in *B. subtilis* biofilms is supported by our observation that *eps* or *tasA* mutants grown in sucrose-based SYM media had a less pronounced defect in biofilm formation compared to the same mutants grown in levan free MSgg or Czapek medium. This suggests that the presence of sucrose and consequently levan in the biofilm EPS may have important functional consequences. Indeed, when *B. subtilis* biofilms were grown in MSgg or Czapek supplemented with sucrose, their resistance to shear stress increased.

While levan contributes to biofilm physico-chemical properties it is not essential for biofilm formation. Biofilms form without levan. In media without sucrose EpsA-O polysaccharide dominates the biofilm matrix. The results of expression of *epsA-O* indicate that the expression levels were similar in SYM and MSgg, suggesting that the concentration of the EpsA-O polysaccharide produced per cell is similar in both biofilms. However, it is likely that EpsA-O represents the major fraction of exopolysaccharides in MSgg, but only a small fraction in levan dominated SYM biofilm. For example, the total mass of EPS per cell is 50-fold higher in SYM-grown biofilms than in MSgg-grown biofilms further strengthening the conclusion that levan comprises the major polysaccharide fraction. However, the strong effect of *eps* knockout would argue that levan is not the primary glue that holds cells together in the biofilm. This is likely attributed to the chemically different EpsA-O polysaccharide.

It is interesting to note that levan is a water soluble polysaccharide [66] with an unusually low viscosity [67]. At concentrations where typical polysaccharides display adhesive behavior by forming gels or pastes, levan solutions remain fluid [66], which would suggest that levan by itself cannot form a strong and sticky network of a typical biofilm. This is in agreement with our observation that significant amounts of water-soluble non-sticky levan were detected in the SYM spent medium below the biofilms. On the other hand, our results clearly show that levan is incorporated into biofilms. Therefore, a mechanism that explains why highly water-soluble levan is retained in the biofilm needs to be proposed. It is possible that TasA or EpsA-O polysaccharides that are already part of the biofilm matrix form a scaffold that retains levan in biofilms. If both *epsA-O* and *tasA* are deleted *B. subtilis* does not develop biofilms irrespective of levan presence. However, if only one was deleted biofilms with levan formed. This supports the conclusion that retention of levan in the *B. subtilis* biofilms is dependent on either protein TasA or EpsA-O polysaccharide that serve as a scaffold for levan entanglement. However, further studies are needed to test this prediction. It is known, however, that soluble and uncharged polysaccharides can interact with proteins or other EPS components in an associative way [52], [58], [68]–[70] thus forming less soluble structures.

Sucrose, which is required for levan formation, is produced by plants and secreted as the root exudate into the rhizosphere. It has been estimated that nearly 5 to 21% of all photosynthetically fixed carbon is transferred to the rhizosphere through root exudates [71]. For example, wheat plants cultivated in mineral medium excrete up to 2.8% of their dry weight in the form of sucrose, fructose and glucose, sucrose being the main component [40]. It is known that *B. subtilis* forms biofilms on plant roots [37]–[39] and *B. subtilis* has often been isolated from the rhizosphere [37], [39], [72] or soil [73], [74]. *B. subtilis* can be used as a plant growth promoting bacterium and as a biopesticide against various pathogens [37]. Therefore an ability to transform sucrose via levanosucrase to levan, which strengthens the biofilm, may increase its competitive advantage in the rhizosphere.

In addition, converting sucrose to levan may provide an additional mechanism to sequester carbon in a highly competitive rhizosphere environment. Levan, entangled in the biofilm, will diffuse away from cells significantly slower than a small molecule of sucrose and may thus serve as carbon storage for *B. subtilis*. Storage carbohydrates such as levan can be selectively hydrolyzed by levanase excreted by *B. subtilis* when other more readily metabolized carbohydrate sources are exhausted [75]. Released sucrose is then transported to the cell interior where intracellular sucrose-6-phosphate hydrolase converts it to key intermediates, glucose and fructose. This may delay sporulation of *B. subtilis*. Indeed, in sucrose-rich SYM medium spores appeared in biofilms significantly later than in Czapek or MSgg grown biofilms. We speculate that levan entanglement in biofilm EPS may prove to be a good strategy for *B. subtilis* when an abundant source of sucrose is available in the environment providing carbon storage for times of famine. However, if this is indeed a mechanism of carbon storage *in situ* remains to be elucidated.

In conclusion, this study showed that *B. subtilis* strain NCBI 3610 is capable of forming floating biofilms in different growth media and that the chemical composition of the medium influences the chemical composition of the biofilm matrix (EPS), biofilm morphology and resistance to shear stress. Furthermore, we showed for the first time that *B. subtilis* produces levan in SYM and sucrose enriched MSgg and Czapeck media and that this polymer is part of the biofilm matrix. In addition, sucrose was able to partially complement the biofilm defective phenotype of *tasA* and *eps* mutants and inclusion of levan in EPS correlated with increased biofilm stability, possibly through entanglement with other matrix components.

## Supporting Information

Figure S1 **Thin-layer chromatogram of hydrolyzed exopolymeric substances (EPS) isolated from biofilms grown in different growth media without sucrose (Czapek, MSgg) and with sucrose.** SYM, Czapek + suc and MSgg + suc. For reference the hydrolyzed levan standard (Levan stand.) from *Erwinia herbicola* is given.
(TIFF)

Figure S2 **Images of biofilms (pellicles) of** ***B. subtilis*** **mutants grown at 37°C for 24 h in petri dishes in SYM, Czapek and MSgg media.** Only mutants (*eps* or *tasA*) that formed biofilms are shown. The scale bar corresponds to 10 mm.
(TIF)

Figure S3 **Images of** ***B. subtilis*** **biofilms (pellicles) grown at 37°C in petri dishes in media without (Czapek and MSgg) and with sucrose.** (SYM, Czapek + sucrose and MSgg + sucrose). The scale bar corresponds to 10 mm.
(TIF)

Figure S4 **Biofilm (pellicle) thickness at different time points during growth in SYM growth medium.** The errors represent standard deviation of the mean (n≥4).
(TIF)
