# Supplementary figures and images for: Exopolymer Diversity and the Role of Levan in Bacillus subtilis Biofilms
Source: PLoS One. 2013 Apr 26;8(4):e62044. doi: 10.1371/journal.pone.0062044 (PMC3637382; doi:10.1371/journal.pone.0062044)

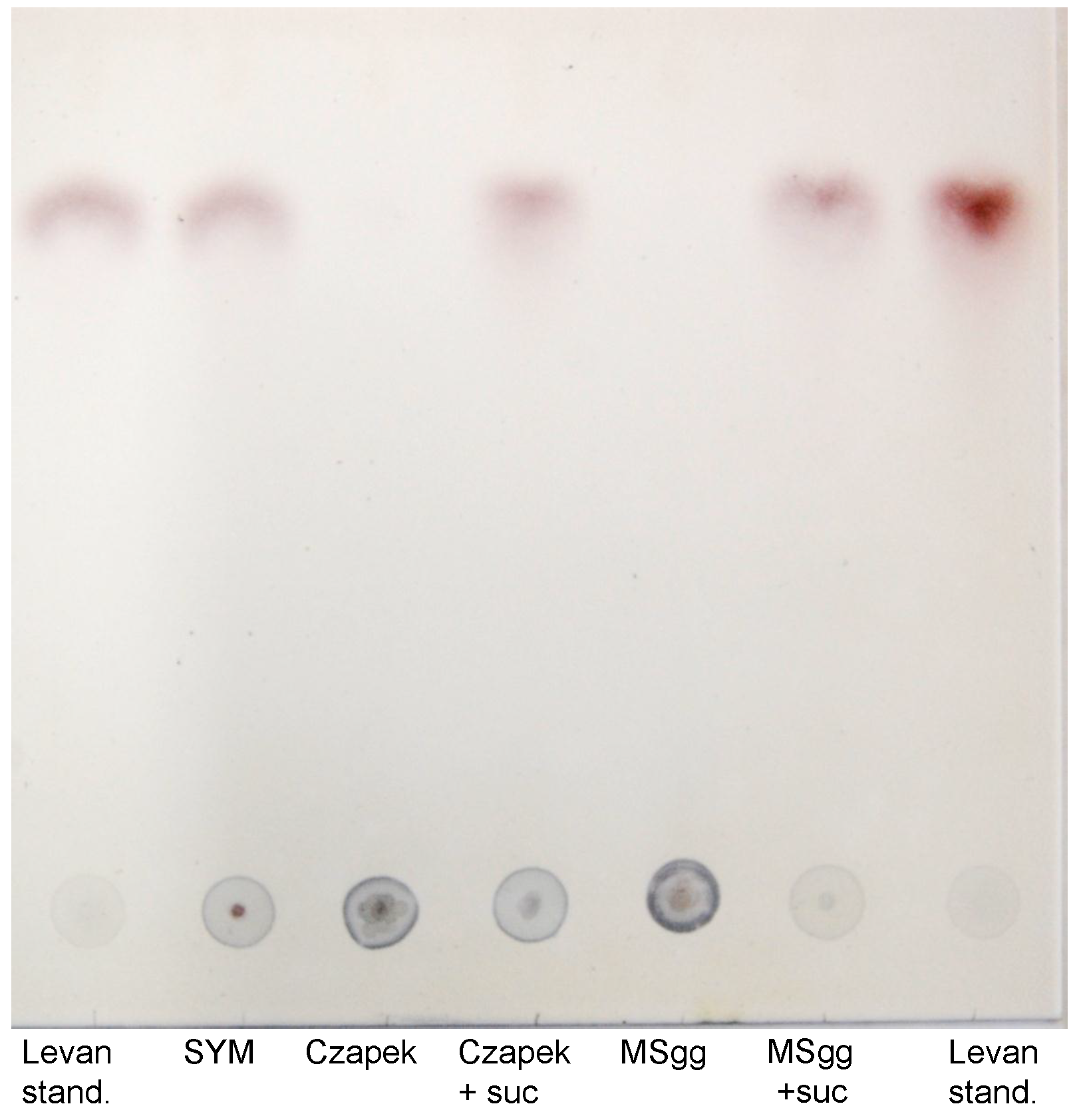

Supplement: Figure S1 — Thin-layer chromatogram of hydrolyzed exopolymeric substances (EPS) isolated from biofilms grown in different growth media without sucrose (Czapek, MSgg) and with sucrose. SYM, Czapek + suc and MSgg + suc. For reference the hydrolyzed levan standard (Levan stand.) from Erwinia herbicola is given. (TIFF) [file pone.0062044.s001.tif]

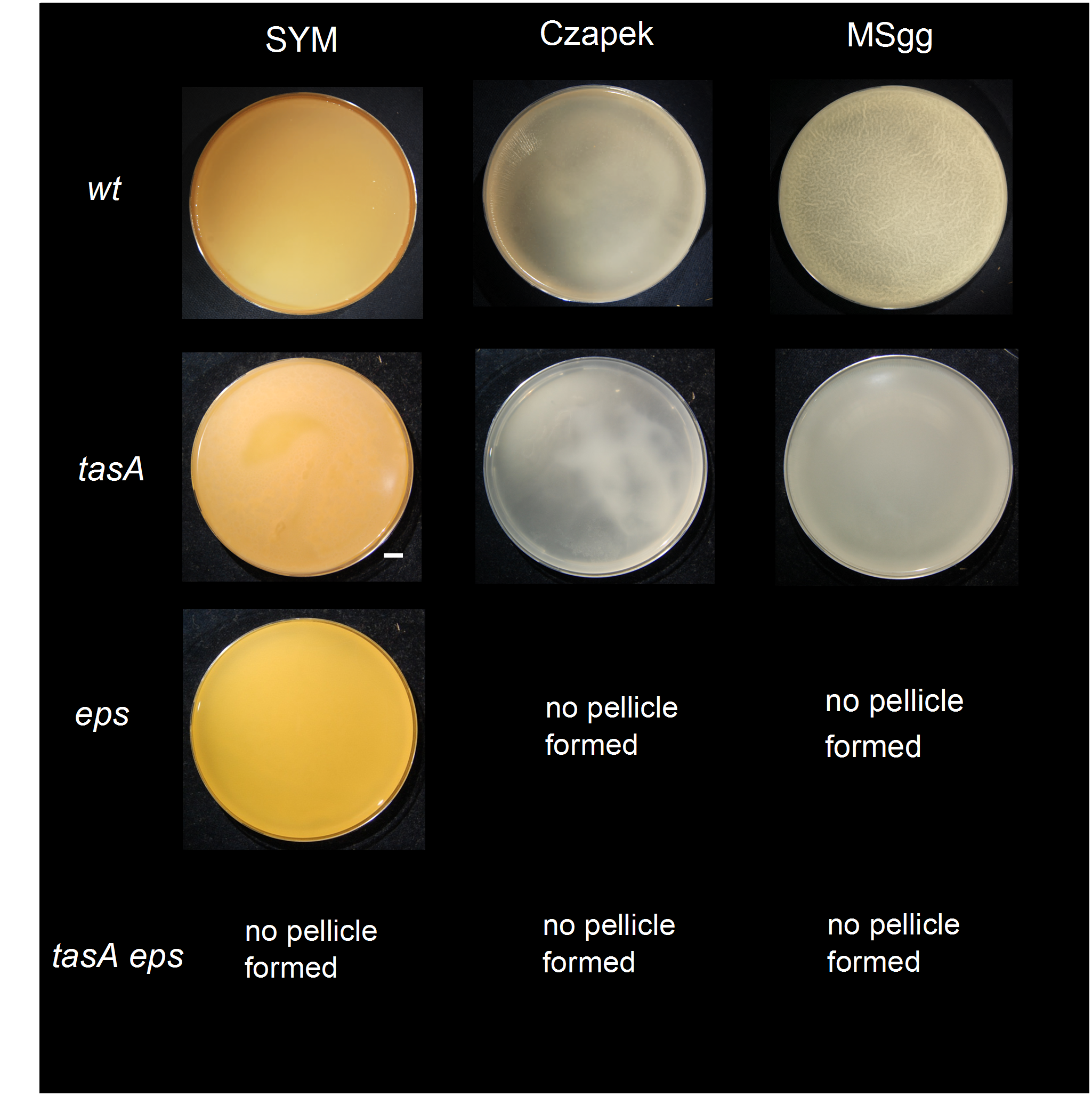

Supplement: Figure S2 — Images of biofilms (pellicles) of B. subtilis mutants grown at 37°C for 24 h in petri dishes in SYM, Czapek and MSgg media. Only mutants (eps or tasA) that formed biofilms are shown. The scale bar corresponds to 10 mm. (TIF) [file pone.0062044.s002.tif]

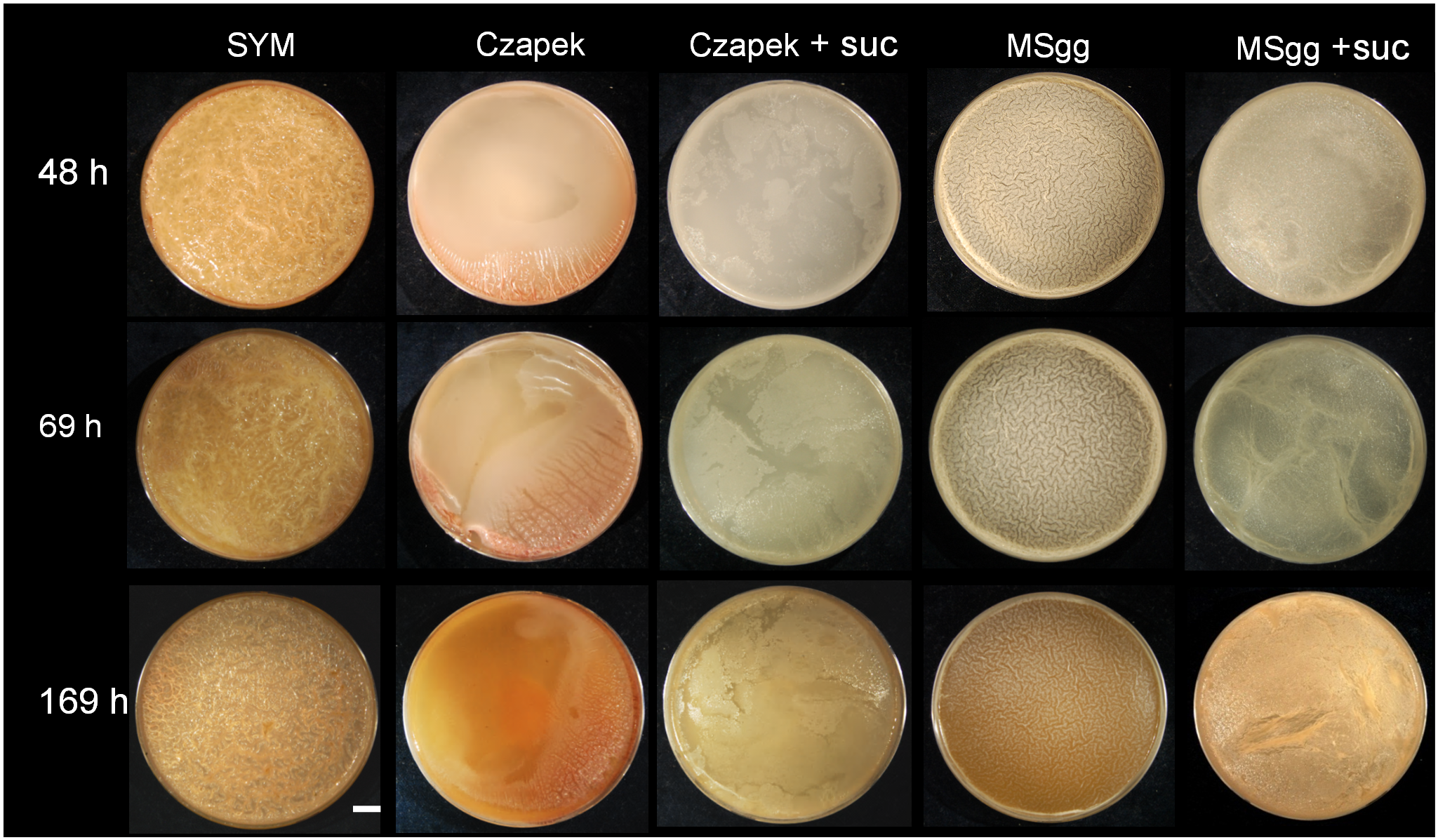

Supplement: Figure S3 — Images of B. subtilis biofilms (pellicles) grown at 37°C in petri dishes in media without (Czapek and MSgg) and with sucrose. (SYM, Czapek + sucrose and MSgg + sucrose). The scale bar corresponds to 10 mm. (TIF) [file pone.0062044.s003.tif]

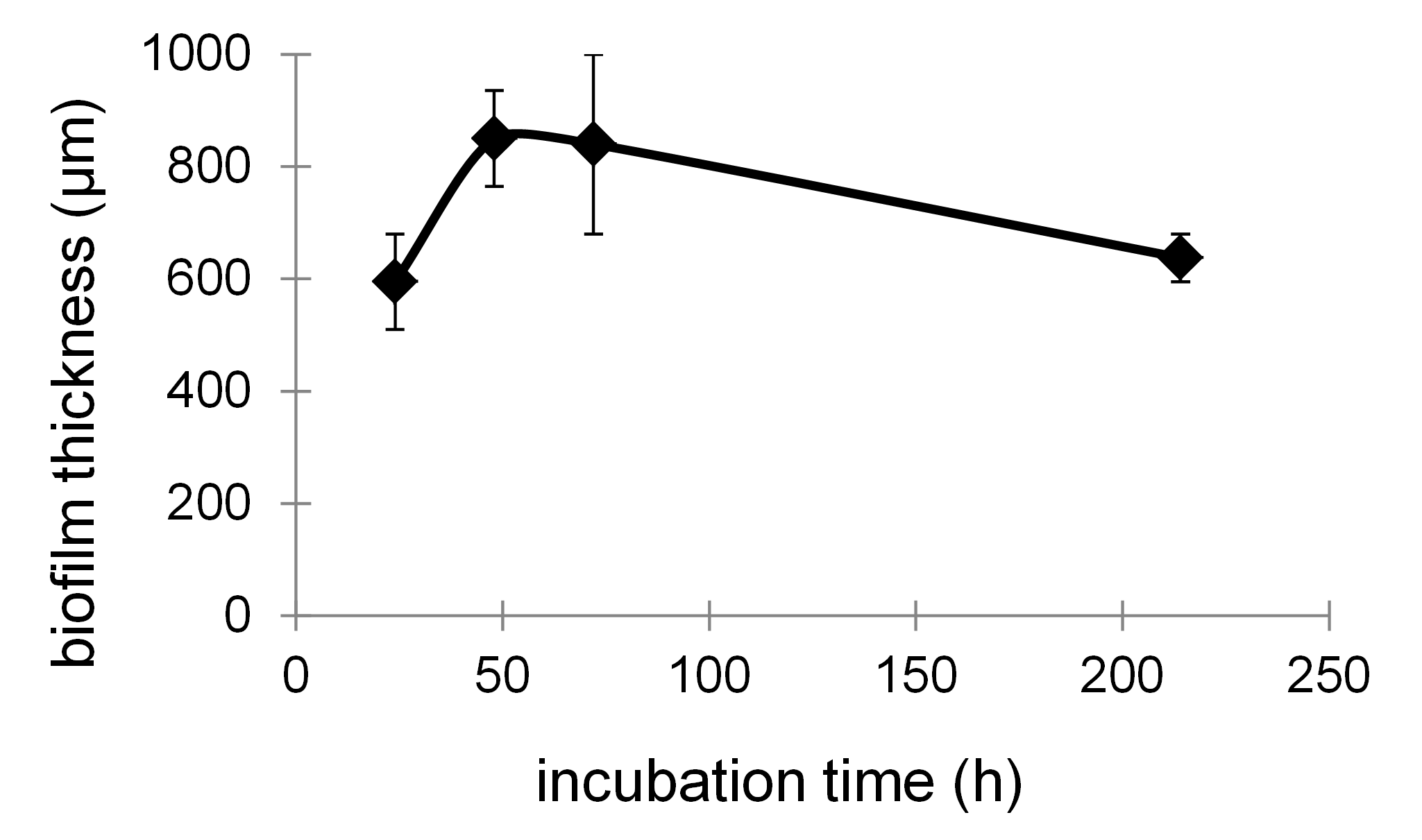

Supplement: Figure S4 — Biofilm (pellicle) thickness at different time points during growth in SYM growth medium. The errors represent standard deviation of the mean (n≥4). (TIF) [file pone.0062044.s004.tif]
